# Topical Application of a Platelet Activating Factor Receptor Agonist Suppresses Phorbol Ester-Induced Acute and Chronic Inflammation and Has Cancer Chemopreventive Activity in Mouse Skin

**DOI:** 10.1371/journal.pone.0111608

**Published:** 2014-11-06

**Authors:** Ravi P. Sahu, Samin Rezania, Jesus A. Ocana, Sonia C. DaSilva-Arnold, Joshua R. Bradish, Justin D. Richey, Simon J. Warren, Badri Rashid, Jeffrey B. Travers, Raymond L. Konger

**Affiliations:** 1 Departments of Pathology & Laboratory Medicine, Indiana University School of Medicine, Indianapolis, IN, 46202, United States of America; 2 Department of Dermatology, Indiana University School of Medicine, Indianapolis, IN, 46202, United States of America; 3 Richard L. Roudebush Veterans Administration Medical Center, Indianapolis, IN, 46202, United States of America; University of Leuven, Rega Institute, Belgium

## Abstract

Platelet activating factor (PAF) has long been associated with acute edema and inflammatory responses. PAF acts by binding to a specific G-protein coupled receptor (PAF-R, *Ptafr*). However, the role of chronic PAF-R activation on sustained inflammatory responses has been largely ignored. We recently demonstrated that mice lacking the PAF-R (*Ptafr*-/- mice) exhibit increased cutaneous tumorigenesis in response to a two-stage chemical carcinogenesis protocol. *Ptafr*-/- mice also exhibited increased chronic inflammation in response to phorbol ester application. In this present study, we demonstrate that topical application of the non-hydrolysable PAF mimetic (carbamoyl-PAF (CPAF)), exerts a potent, dose-dependent, and short-lived edema response in WT mice, but not *Ptafr* -/- mice or mice deficient in c-*Kit* (c-*Kit* ^W-sh/W-sh^ mice). Using an ear inflammation model, co-administration of topical CPAF treatment resulted in a paradoxical decrease in both acute ear thickness changes associated with a single PMA application, as well as the sustained inflammation associated with chronic repetitive PMA applications. Moreover, mice treated topically with CPAF also exhibited a significant reduction in chemical carcinogenesis. The ability of CPAF to suppress acute and chronic inflammatory changes in response to PMA application(s) was PAF-R dependent, as CPAF had no effect on basal or PMA-induced inflammation in *Ptafr*-/- mice. Moreover, c-*Kit* appears to be necessary for the anti-inflammatory effects of CPAF, as CPAF had no observable effect in c-*Kit* ^W-sh/W-sh^ mice. These data provide additional evidence that PAF-R activation exerts complex immunomodulatory effects in a model of chronic inflammation that is relevant to neoplastic development.

## Introduction

Platelet activating factor (1-alkyl-2-acetyl glycerophosphocholine, PAF) is a bioactive lipid molecule produced by both enzymatic and non-enzymatic mechanisms [1]. Non-enzymatic production of PAF is dependent on free-radical mediated modification of the sn-2 polyunsaturated fatty acid of glycerophosphocholines to form PAF itself and other oxidized glycerophosphocholines (ox-GPCs) that exhibit the ability to activate the PAF receptor [1]–[5]. The biological activity of PAF is mediated by binding to a single G-protein coupled receptor (PAF-R) that is expressed on a wide variety of cells including keratinocytes [1], [6], [7]. Once produced, PAF elicits a variety of physiological and pathological effects that play a role in acute inflammation, wound healing, and angiogenesis. However, PAF is perhaps best known for its pro-inflammatory effects that mediate the systemic reaction to shock, allergic reactions, and anaphylaxis [1]. In this role, PAF's actions are thought to be mediated by its ability to stimulate vasodilation and vascular permeability, platelet aggregation, bronchoconstriction, and alterations in leukocyte function [1]. These effects on leukocytes include the ability of PAF to stimulate mast cell activation and migration [8]–[10], mononuclear and neutrophilic phagocytosis [11]–[13], and M2 polarization of macrophages [14].

While it is clear that PAF acts to promote acute inflammatory effects, particularly those associated with anaphylaxis and shock, more recent evidence suggests that PAF may play a more complex role in immune function. PAF production through enzymatic and oxidative pathways appears to play a role in keratinocyte responses to genotoxic agents, such as ultraviolet B, cigarette smoke or chemotherapeutic agents [3], [15]–[18]. Studies on the mechanisms of ultraviolet B (UVB)-induced systemic immunosuppression have demonstrated a requirement for PAF-R activation [3], [10], [19], [20]. This UVB-induced systemic immunosuppression is characterized by an antigen-specific suppression of adaptive T-cell mediated immune responses [21], [22]. In addition, we have recently demonstrated that *Ptafr*-/- mice exhibit an increase in cutaneous chemical carcinogenesis that is associated with a corresponding increase in phorbol ester (PMA)-induced cutaneous inflammation [7]. This data suggests that PAF-R activation may be also be important in regulating the innate immune system. However, the down-stream cellular mediators of this immunomodulatory activity are unknown.

The c-*Kit* gene codes for a receptor tyrosine kinase that binds the ligand, stem cell factor (SCF), and is important in [23]. The c-*Kit^W-sh^* (sash) mutation is an inversion of a 3.1 Mbp segment that disrupts the promoter region of the c-*Kit* gene [23]. Mice homozygous for the c-*Kit^W-sh^* mutation exhibit a profound loss of mast cells, but also lack melanocytes and interstitial cells of Cajal [23], [24]. Thus, c-Kit^W-sh/W-sh^ mice represent a common tool to assess mast cell function. Mast cells are tissue resident bone marrow derived cells, which like PAF, are well known mediators of allergic and anaphylactic reactions [25]. Mast cells are strategically localized in the subepithelial and submucosal spaces prone to environmental and infectious insults [25]. Moreover, the ability of PAF to promote anaphylactic responses is dependent on mast cell activation [8]. PAF is also a known activator and chemotactic agent for mast cells [9]. In addition to their role in promoting the wheel and flare reaction associated with type I hypersensitivity reactions, mast cells have also been shown to suppress chronic inflammation as well as adaptive immune responses [26]–[31]. Of particular relevance, mast cells act to limit murine models of contact hypersensitivity and chronic UVB-induced inflammation [32]. In addition, the role of the PAF-R in UVB-induced immunosuppression has been shown to result from PAF-R-dependent migration of mast cells to the lymph node, wherein they exert an interleukin (IL)-10-dependent immunosuppressive effect [10].

While we recently provided evidence that PAF has anti-inflammatory effects on chronic PMA-induced tumor promotion [7], it is possible that the effects of germ-line loss of the PAF-R could be due to compensatory embryonic or postnatal alterations in skin development [7]. Thus, a demonstration that PAF-R agonist treatment results in suppression of DMBA/PMA-induced carcinogenesis and inflammation would provide further support to the idea that the PAF-R has important anti-neoplastic and immunomodulatory effects. Our current studies examining the role of the PAF-R in PMA-induced inflammation adds to our previous report and reveals a complex immunomodulatory role for PAF-R signaling. Moreover, our findings using c-*Kit^W-sh/W-sh^* mice demonstrate a role for *c-Kit* and possibly mast cells in the immunomodulatory effects of PAF.

## Methods

### Ethics statement

The protocols were approved by the Committee on the Ethics of Animal Experiments of the Indiana University School of Medicine (Institutional Animal Care and Use Committee (IACUC) (Protocol Number: 3841 and 10639). Every attempt was made to minimize animal suffering.

### Reagents and chemicals

Phorbol 12-myristate 13-acetate (PMA) was purchased from Promega, Madison, WI. 7,12-Dimethylbenz(a)anthracene (DMBA) was obtained from Acros Organics, Fair Lawn, NJ. Carbamyl-PAF was obtained from Sigma-Aldrich (St. Louis, MO).

### Animals

*Ptafr* knockout (*Ptafr*-/-) in the C57BL/6 background were originally obtained from Dr. Satoshi Ishii (Department of Biochemistry and Molecular Biology, Faculty of Medicine, The University of Tokyo), derived as previously described [33]. Age (8–12 week)-matched *Ptafr* +/+ C57BL/6 (WT) were used as controls (The Jackson Laboratories, Bar Harbor, ME). SKH-1 hairless, albino mice were obtained from Charles Rivers (Wilmington, MA). Mice containing the W-sash (W-sh) inversion mutation in the promoter region of the *c-kit* gene (*Kit^W-sh^*) were obtained from the Jackson Laboratories in the C57Bl/6 background (B6.Cg-*Kit^W-sh^*/HNihrJaeBsmGlliJ). Mice were housed under specific pathogen-free conditions at the Indiana University School of Medicine.

### Ear thickness measurements

The left ear of each mouse was treated with 20 µl of cPAF (0.1, 0.3, or 1 mM in acetone). The right ear was treated with 20 µl of vehicle (acetone) alone. For PMA treatments, the left ear was treated with 10 µg of PMA in 20 µl of acetone, and the right ear was treated with vehicle (VEH) alone 3 times a week (arrows) for 16 days. For PMA and CPAF treatments, the mice were treated with PMA first, then 20 µl of 0.3 mM CPAF (in acetone) was added immediately after the PMA treatment. Ear thickness was measured at the indicated time points following treatment using a constant pressure analog thickness gauge (Peacock Model G, 0.4 N).

### Epidermal thickness measurements

Mouse ears were treated for 18 days thrice weekly with vehicle, CPAF (6 nmole), PMA, or PMA and CPAF were formalin fixed and paraffin embedded (FFPE), and stained with hematoxylin & eosin (H&E). Epidermal thickness was measured by measuring the epidermal thickness 10 times in 5 consecutive 200 x fields, starting at the distal tip of the ear and moving proximally to the next adjacent field. Epidermal thickness was quantitated using an eyepiece reticle calibrated with a stage micrometer. All slides were blinded prior to measurement.

### Tumorigenesis studies

For two stage chemical carcinogenesis studies, the dorsal skin of SKH-1 hairless mice were treated with once with DMBA, followed by repetitive treatments with PMA as previously described [7]. For tumor counting, only durable tumors >1 mm in greatest diameter were counted. After 25 weeks of PMA treatment, mice were sacrificed and tumor size (size at greatest dimension) was measured for each tumor. FFPE tumor sections from WT and *Ptafr* -/- mice were stained with H&E and the tumor type (Papilloma and microinvasive SCC (MISCC)) was assessed in blinded fashion by a board-certified dermatopathologist, as previously described [34]. For MPO activity, tumor-free areas of treated skin were removed and MPO activity levels were assessed as previously described [35].

### PMA-induced inflammation in dorsal epidermis

The back skin of female WT or *Ptafr*-/- mice was shaved under anesthesia and was then treated with PMA or vehicle three times a week for 18 days as described above. Immediately after each PMA application, the mice were treated with vehicle or 100 µl of 0.3 mM CPAF (in acetone). On day 18, the mice were sacrificed and the treated dorsal epidermis was excised for skin thickness measurements, as previously described [35].

### Mast cell counting

Mast cells were stained with toluidine blue in deparaffinized FFPE sections as described [36]. Four 200x images per biopsy specimen were captured sequentially along the length of the section using a Nikon Eclipse 80i. Nikon Elements Basic Research Image Analysis software (v. 4.13) was used to count the mast cells and measure the dermal area of interest. The total area and cell counts were then averaged [(mast cells/µm^2^)x10^−5^]. Given that mast cell counts differ at different body sites in mice, mast cell counts were performed in both the ears and the dorsal (back) epidermis.

### Statistical analysis

Statistical significance was assessed by Prism 5.0 software (Graph Pad Software, San Diego CA) and significance was set as *p*<0.05.

## Results

### CPAF is topically active and results in a dose-dependent transient increase in ear thickness that is dependent on mast cells

**Figure 1:**
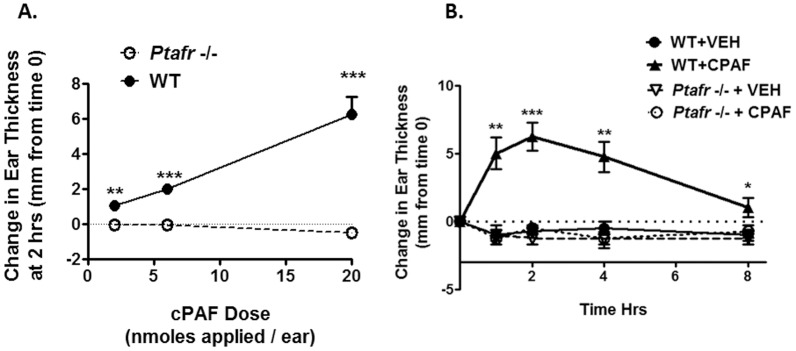
Dose and time related acute ear inflammation changes in response to topical CPAF. *1A*. *Topical CPAF dose-dependently induces rapid inflammatory responses as measured by ear thickness measurements.* One ear of WT and *Ptafr* (-/-) were treated with one of three doses of CPAF (20 µl of a 0.1, 0.3, and 1.0 mM solution for a total treatment dose of 2, 6, or 20 nmole CPAF per ear). The contralateral ear was treated with acetone alone (VEH). Ear thickness was measured prior to treatment and 2 hours after treatment. After the pretreatment ear thickness values were subtracted, the mean and SEM were plotted (n = 4 for 20 nmole and n = 8 for 2 & 6 nmole CPAF & VEH treated mouse ears). *1B*. *Topical CPAF treatment induces a rapid, but transient increase in inflammation as measured by ear thickness changes.* One ear of wildtype (WT) and *Ptafr* (-/-) mice was treated with 20 µl of CPAF (20 nmoles of a 0.1 mM solution in acetone) and 20 µl of acetone (VEH) was applied to the contralateral ear. Ear thickness was measured just prior to reagent application and at 1, 2, 4, and 8 hours after application. Results represent the mean and SEM (n = 4 mice) after subtracting the pretreatment ear thickness. CPAF induced a significant increase in ear thickness in WT mice relative to the WT+VEH treated ears. *, *p*<0.05; **, *p*<0.01; ***; *p*<0.001; 2-tailed *t*-test.

In a previous study [7] and in Fig 1A, we show that a single topical application of increasing doses of the non-hydrolyzable PAF-R agonist, CPAF, results in a dose-dependent increase in ear thickness at two hours after application. In Fig 1B, a time course study demonstrates that CPAF-induced ear thickness changes occur rapidly, with significant increases in ear thickness noted by 1 hour after application and peak ear thickness was noted at 2 hrs. Importantly, following a single application of CPAF the changes in ear thickness were transient and declined to near baseline by 8 hours after application. The rapid and transient nature of the response strongly suggests that changes in vascular permeability leading to edema formation are largely responsible for the changes in ear thickness. This is consistent with the well-known ability of PAF to induce vasodilation and vascular permeability [37]. To rule out PAF-R independent effects [38], mice with germline loss of the *Ptafr* gene (*Ptafr*-/- mice) were also treated with CPAF. At all doses studied, topical CPAF application failed to elicit an inflammatory reaction in *Ptafr*-/- mice (Figs 1A&B), verifying the specificity of the pharmacological effect.

**Figure 2:**
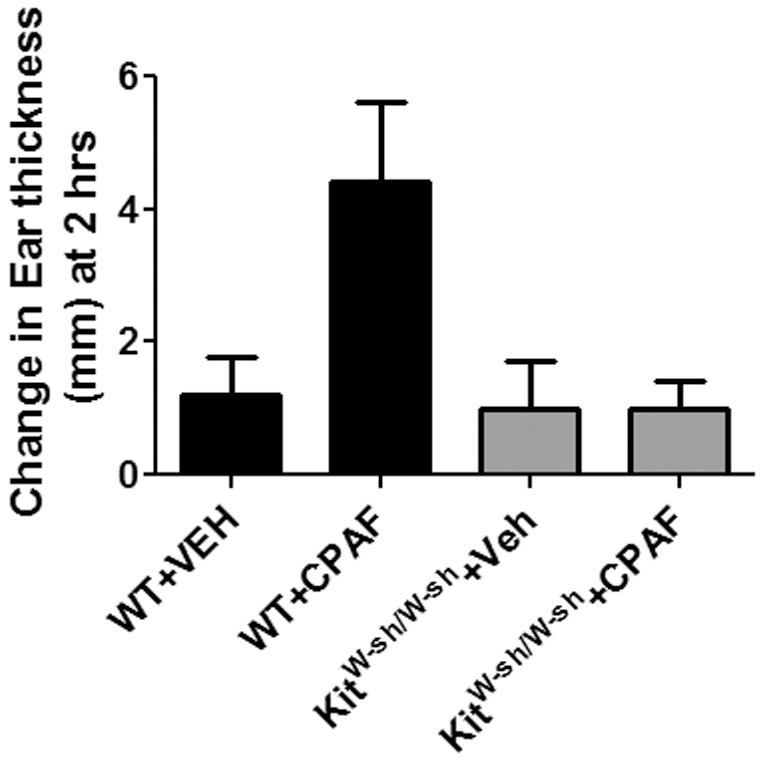
CPAF induces transient ear thickness changes are blocked in c-Kit^W-sh/W-sh^ mast cell deficient mice. WT and c-Kit^W-sh/W-sh^ mice were treated with vehicle (VEH) alone on one ear, and 20 ml of 0.3 mM CPAF (6 nmole) on the contralateral ear. Ear thickness was measured both prior to and 2 hrs after reagent application. After subtraction of the ear thickness at time 0, the mean and SEM were plotted (n = 5 for WT mice, n = 4 for Kit^W-sh/W-sh^ mice). *, *p*<0.05; 2-tailed *t*-test.

Finally, the ability of CPAF to induce rapid edema reactions has been linked to mast cell activation [8], [9]. We therefore determined whether the ability of CPAF to induce an early edema reaction at 2 hrs could be blocked in mice lacking mast cells (c-Kit^W-sh/W-sh^ mice). In Fig 2 we verify that mast cells are indeed required for the early increase in ear thickness mediated by CPAF application.

### Topical CPAF protects against cutaneous chemical carcinogenesis

The studies in Fig 1 demonstrate that CPAF exhibits specific dose-dependent pharmacologic activity following topical application. The use of a topical delivery approach also avoids potential toxicities associated with systemic PAF-R agonist treatment [37]. In addition, the PAF-R is prone to the phenomenon of ligand-induced down-regulation and desensitization [1], [39]; this regulatory feature is characteristic of G-protein coupled receptors and can lead to a paradoxical loss of receptor activation by subsequent ligand binding [1], [40]. Thus, given that carcinogenesis studies would require long-term repetitive application of CPAF, we utilized the 0.3 mM concentration of CPAF which induced an intermediate, but significant increase in ear thickness measurements (Fig 1A). For these studies, CPAF was applied immediately after DMBA or PMA application for the duration of the study.

**Figure 3:**
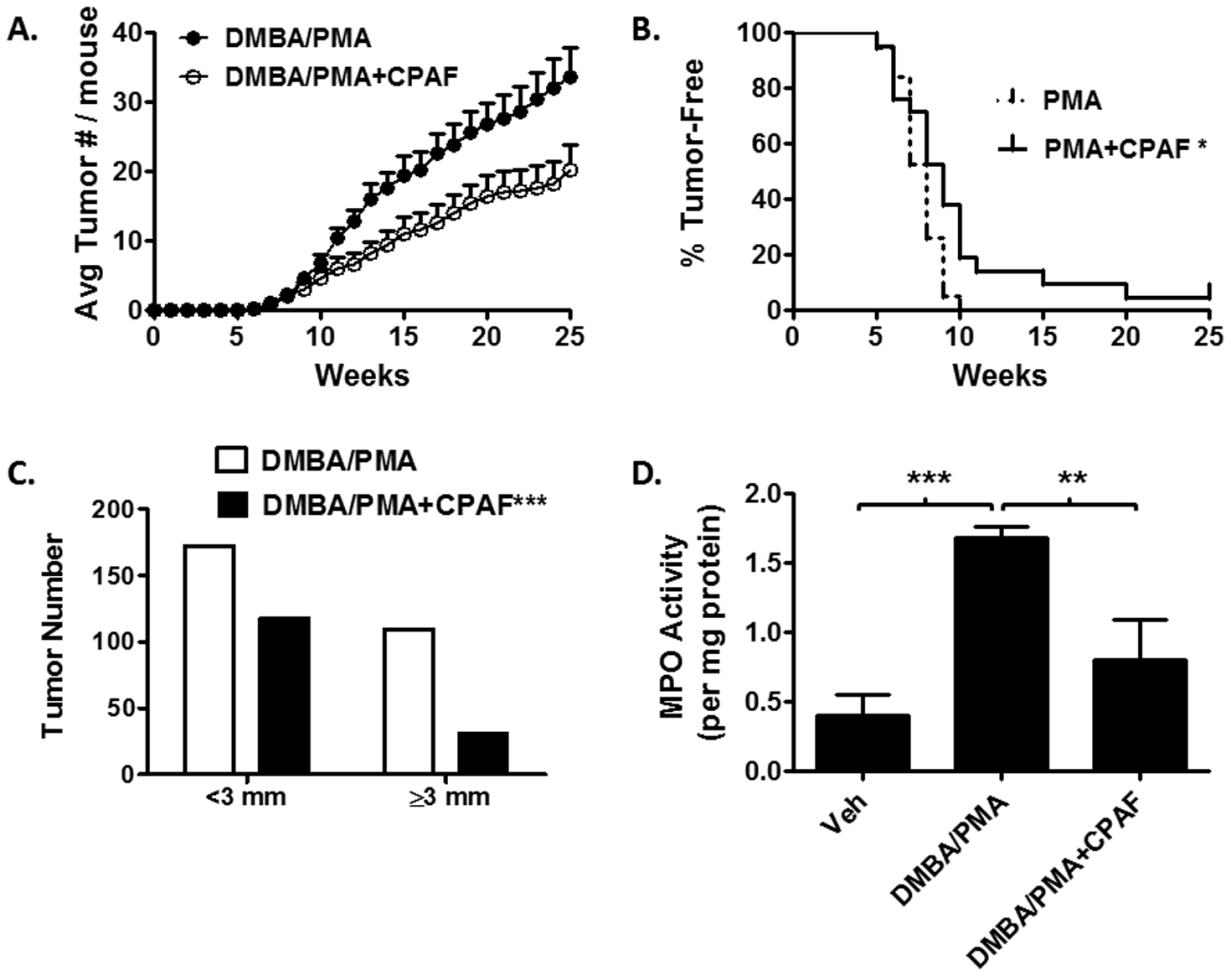
Topical application of CPAF suppresses DMBA/PMA-induced tumorigenesis. *3A. Topical treatment with CPAF suppresses DMBA/PMA-induced tumor multiplicity.* SKH-1 mice were treated once with DMBA+/- CPAF, then with PMA or PMA+CPAF for 25 weeks. Durable tumors were counted on a weekly basis. Tumor multiplicity (Avg tumor number per mouse was plotted at each week. Results represent the mean and SEM for n = 19–20 mice/group. *p*<0.05 for weeks 9–12,14–25; *p*<0.01 for week 13; Mann-Whitney U test. *3B. Topical CPAF delayed tumor incidence in mice treated with DMBA/PMA.* The percent of mice remaining tumor free over the 25 week chemical tumorigenesis study were plotted using a survival curve. The treatment with CPAF resulted in a significant change in the tumor incidence, with a median time until first tumor occurrence of 8 weeks for DMBA/PMA treated and 9 weeks for DMBA/PMA+CPAF treated mice. *, *p*<0.05; Log-rank (Mantel-Cox) Test. *3C*. *Topical CPAF treatment results in a smaller number of large tumors (≥3 mm in greatest diameter) after 25 weeks of treatment.* Tumor size distribution was plotted as the number of tumors in each size distribution for each treatment group. Histopathologic exam showed no significant difference in the rates of papilloma and SCC formation between the treatment groups (not shown). ***, *p* = 0.0001 Fisher's exact test. *3D*. *DMBA/PMA-induced MPO activity is suppressed by CPAF.* After the mice were euthanized following 25 weeks of DMBA/PMA +/- CPAF treatment, tumor free areas of skin were excised and MPO activity was assessed in tissue lysates. After normalization to total protein, MPO activity was plotted as the mean and SEM (n = 5–9 mice per group). **, *p*<0.05; ***, *p*<0.001; 2-tailed *t*-test.

Consistent with our previous study that demonstrated that *Ptafr*-/- mice exhibit increased DMBA/PMA-induced tumor formation, in Fig 3A we show that topical treatment of CPAF resulted in a significant reduction in DMBA/PMA-induced tumor burden. CPAF treatment also demonstrated a modest, but significant protective effect on tumor incidence (Fig 3B). Moreover, while *Ptafr*-/- mice exhibited a significant increase in the frequency of larger tumors [7], mice treated with topical CPAF exhibited a significant increase in smaller tumor sizes (Fig 3C). In addition, we previously demonstrated that *Ptafr*-/- mice exhibit an increase in SCC formation relative to WT mice [7]. However, in this current study, no frank SCCs were observed and mice treated topically failed to exhibit a significant change in the proportion of grade 1–3 papillomas or microinvasive SCCs (Figure S1 in File S1). Finally, tumor-free areas of skin treated with CPAF exhibited a marked reduction in DMBA/PMA-induced inflammation, as assessed using myeloperoxidase (MPO activity), a marker of granulocytic cell infiltrates (Fig 3D) [41]. This is consistent with our previous results in *-/-* mice, which exhibited an augmentation of MPO activity following chronic, repetitive PMA treatments [7].

### Topical CPAF treatment suppresses inflammation induced by repeated applications of the tumor promoter PMA

**Figure 4:**
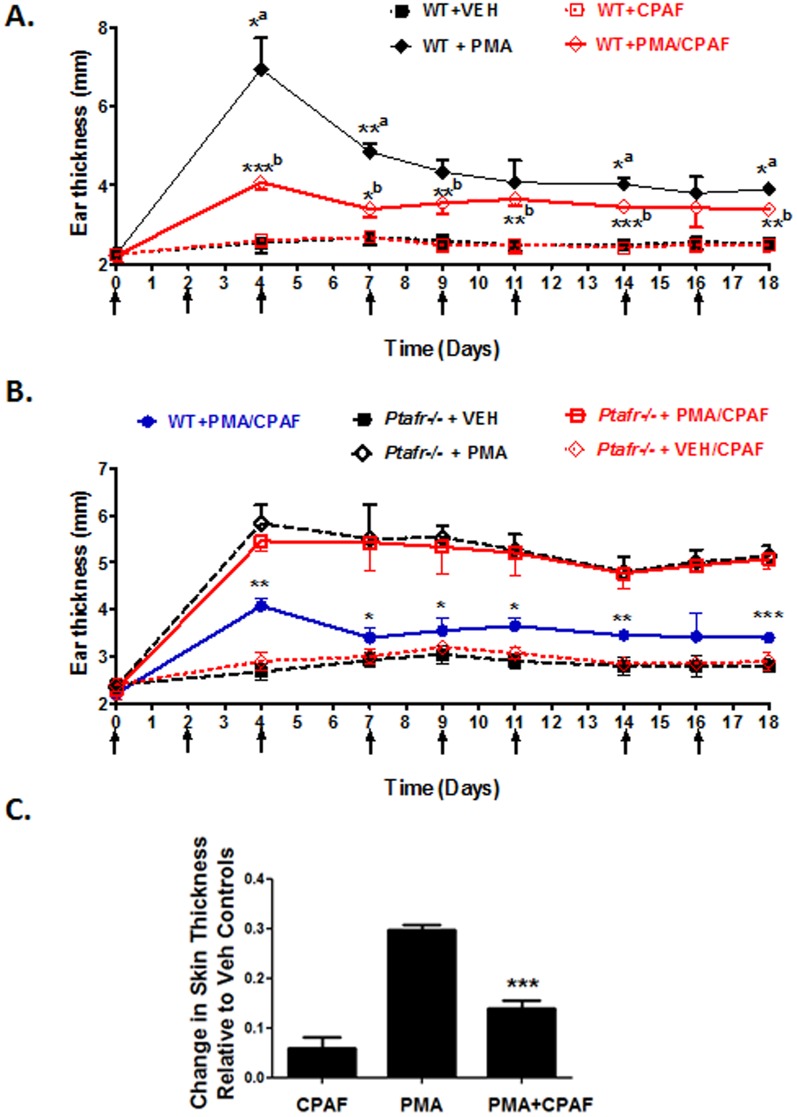
Effect of CPAF treatment on ear thickness changes over an 18 day course of thrice weekly PMA applications in WT and *Ptafr*-/- mice. *4A. Topical CPAF treatment suppresses PMA-induced changes in ear thickness in WT mice.* CPAF (6 nmoles) alone or PMA +/- CPAF were applied to WT mouse ears thrice weekly for 18 days. Skin thickness measurements were taken at time 0 and just prior to each reagent application. After subtraction of the time 0 ear thickness, ear thickness changes were plotted as the mean and SEM (n = 4–5 mice per group). PMA relative to PMA+CPAF (^a^), PMA+CPAF relative to CPAF(^b^); *, *p*<0.05; **, *p*<0.01; ***, *p*<0.001; 2-tailed *t*-test. *4B. Topical CPAF treatment is ineffective in altering PMA-induced ear thickness changes in Ptafr-/- mice. Ptafr*-/- mice were treated and assessed as in 4A above. For the sake of comparison, the data for the PMA + CPAF treatment in WT mice is included. (mean and SEM; n = 4–5 mice per group). WT mice treated with PMA + CPAF exhibit a significant decrease in ear thickness measurements relative to *Ptafr*-/- mice treated with PMA + CPAF (*, *p*<0.05; **, *p*<0.01; ***, *p*<0.001; 2-tailed *t*-test). *4C. Topical CPAF treatment suppresses PMA-induced skin thickness increases in dorsal epidermis following 18 days of treatment.* The dorsal epidermis of SKH-1 mice was treated thrice weekly with vehicle, CPAF, PMA, or PMA + CPAF. Doses of PMA and CPAF were the same as that used for the tumorigenesis studies in Fig 3. ***, *p*<0.05 relative to PMA treated skin; 2-tailed *t*-test (n = 3 per group).

In our previous report [7], we showed that a first PMA application induced a significant increase in ear thickness in WT mice 2 days after application. A second PMA application resulted in a further increase in ear thickness that partially resolved. Thereafter, subsequent PMA applications failed to elicit further increases in ear thickness. In contrast, a significant and persistent increase in ear thickness was observed during the 18 days of PMA treatments (sustained chronic inflammatory phase). As in our previous study, loss of the PAF-R resulted in a blunting of the peak ear thickness observed after the second PMA application, but a higher level of sustained chronic inflammation after continued thrice weekly PMA applications (Figure S2 in File S1 and [7]). We therefore examined whether topical CPAF administration would have the opposite effect and suppress the sustained chronic inflammation induced by PMA treatment. In Fig 4A we show that the application of topical CPAF immediately after each application of PMA results in a significant reduction in both the peak inflammatory response noted on day 4, as well as a reduction of the sustained inflammatory response that was noted throughout the period of PMA application (18 days). Interestingly, at these later time points, CPAF had no effect on ear thickness measurements in WT mice not treated with PMA, suggesting that topical CPAF is ineffective in inducing a significant sustained inflammatory response at the dose tested (0.3 mM). Finally, we next demonstrated that the effect of CPAF was entirely dependent on the PAF-R, as CPAF and Veh applications showed nearly identical ear thickness measurements at all time points following PMA application to *Ptafr-/-* mice (Fig 4B). While CPAF significantly blocked PMA-induced ear thickness in WT mice (Fig 4A), this response was completely lost in *Ptafr*-/- mice treated with PMA (Fig 4B). Finally, in Fig 4C, we show that the ability of CPAF to suppress PMA-induced skin thickness changes was not dependent on anatomic site. CPAF significantly suppressed PMA-induced increases in dorsal back skin thickness after 18 days of thrice weekly treatment.

**Figure 5:**
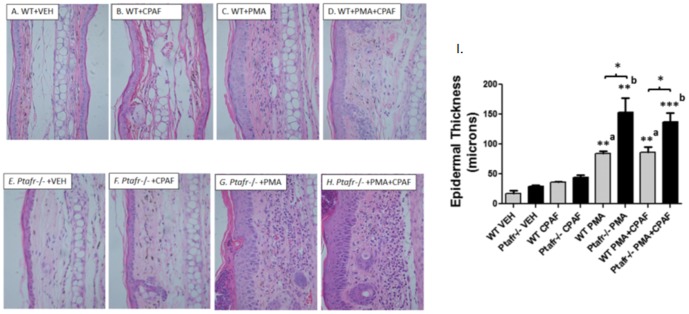
Inflammatory infiltrates and epidermal thickness in WT and *Ptafr*-/- mouse ears treated chronically with CPAF, PMA or PMA + CPAF. *5A*–*H. Inflammatory infiltrates are increased in Ptafr-/- mice treated with PMA, while CPAF suppresses inflammatory cell infiltrates in PMA treated WT mice.* After treating WT and *Ptafr*-/- mouse ears for 18 days with VEH, CPAF, PMA, or PMA + CPAF as described in Fig 4, mice were sacrificed and the ears excised for formalin fixation, paraffin embedding and hematoxylin & eosin (H&E) staining. Photomicrographs taken at 400x magnification are shown. *5I. PMA-induced epidermal thickness is augmented in Ptafr-/- mice, while CPAF has no effect on PMA-induced epidermal thickness in either WT or Ptafr-/- mice.* Epidermal thickness was measured in WT and *Ptafr*-/- mice treated for 18 days with VEH, CPAF, PMA, and PMA + CPAF as described in Fig 4A&B. Epidermal thickness in mm is expressed in the mean and SEM for 3–4 ears per experimental group. Significantly different from WT VEH control (^a^), significantly different from the *Ptafr*-/- VEH control (^b^). *, *p*<0.05; **, *p*<0.01; ***, *p*<0.001; 2-tailed *t*-test.

We next determined the histopathologic changes in ear skin that result from multiple PMA applications with or without CPAF treatments. Multiple PMA applications to mouse skin are known to induce epidermal hyperplasia and leukocyte infiltration [42], [43]. In Fig 5A–I, we show that multiple PMA applications to WT mouse ears results in a clear increase in ear thickness accompanied by dermal expansion, leukocyte infiltration and epidermal hyperplasia. Moreover, co-treatment of WT mice ears with CPAF resulted in a reduction in PMA-induced leukocyte infiltrates while *Ptafr*-/- mice exhibited a marked increase in ear thickness, dermal expansion, inflammatory cell infiltrates, and epidermal hyperplasia (Fig 5A–H). Relative to WT mice, *Ptafr*-/- mice exhibited a marked increase in granulocytic infiltrates following chronic PMA treatments (Fig 5 and Figure S3 in File S1). Moreover, CPAF treatment suppressed the observed PMA-induced inflammatory infiltrates in WT, but not *Ptafr*-/- mice. This data is in agreement with our previous study demonstrating that *Ptafr*-/- mice exhibit a marked increase in MPO activity following PMA treatment [7], as well as our data in Fig 3D that shows that CPAF treatment suppresses MPO activity in DMBA/PMA treated mice. Since neutrophils are shown to play an important role in mouse skin photocarcinogenesis [35], this reduction in granulocytic infiltrates could play an important role in the observed anti-tumor effects of topical CPAF treatment in chemical carcinogenesis (Fig 3A&B). Given that the degree of chronic inflammation is commonly associated with epidermal hyperplasia, it is somewhat surprising that while CPAF treatment suppressed PMA-induced granulocytic inflammation in WT mice, CPAF treatment did not alter PMA-induced epidermal hyperplasia in either WT or *Ptafr-/-* mice (Fig 5I).

### PAF exerts complex effects on the initial inflammatory response to PMA application

**Figure 6:**
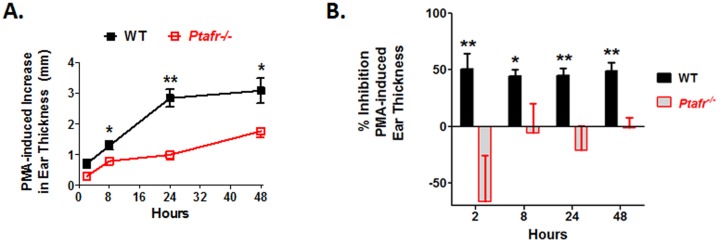
Following a single application of PMA, *Ptafr-*/- mice exhibit a reduction in PMA-induced ear thickness while CPAF treatment also induces a paradoxical PAF-R dependent decrease in PMA-induced ear thickness. For all studies in Figs 6, WT and *Ptafr*-/- mice were treated with VEH, PMA, CPAF (6 nmole) or PMA + CPAF as described in Fig 4A&B. Ear thickness measurements were made just prior to reagent application as well as at the indicated time points up to 48 hrs. *6A. Ptafr-/- mice exhibit a reduction in early and delayed ear thickness increases following a single PMA application.* Ear thickness plots for WT and *Ptafr*-/- mice treated with and without PMA are shown. Statistically significant changes are noted to WT mice treated with PMA relative to *Ptafr*-/- mice treated with PMA. Results represent the mean and SEM of ear thickness after subtracting the ear thickness at time 0 (n = 4–12 mice per group). *, *p*<0.05; **, *p*<0.01; *t*-test. *6B. Coadministration of topical CPAF blocks PMA-induced increases in ear thickness at all time points (2–48 hrs).* PMA-induced ear thickness changes were assessed in WT or *Ptafr*-/- mice treated with PMA or PMA + CPAF. After subtracting the ear thickness at time 0, the ability of CPAF to suppress PMA-induced ear thickness changes was calculated as a percentage inhibition of PMA-induced ear thickness increases. CPAF treatment resulted in a significant inhibition of PMA-induced ear thickness changes at all time points (p<0.01–0.05; % inhibition significantly different from no inhibition, Wilcoxon Signed Rank Test). CPAF treatment had no significant effect on PMA-induced ear thickness changes in *Ptafr*-/- mice (One sample analysis, Wilcoxon Signed Rank Test). The percent inhibition of PMA-induced ear thickness changes by CPAF in WT mice was also significantly different than that seen in *Ptafr*-/- mice (*, *p*<0.05; **, *p*<0.01; *t*-test).

The pro-inflammatory tumor promoter PMA is known to induce a rapid edema reaction in mouse ears [42], [43]. This is followed by a gradual rise in leukocyte infiltration as measured by MPO and/or N-acetylglucosaminidase (NAF) activity that peaks at around 24 hrs [42], [43]. Given that PAF is a potent vasoactive lipid mediator that promotes rapid edema reactions [37], we next examined how CPAF would affect early inflammatory changes following the first PMA application in WT and *Ptafr*-/- mice. As seen in Fig 6A, PMA treatment induces a rapid increase in ear thickness that was first observed two hours after application. Ear thickness changes peaked at approximately 24 to 48 hrs. In contrast, *Ptafr*-/- mice exhibited a reduction in the initial ear thickness changes noted at 2 & 8 hrs, suggesting that PMA-induced edema responses were PAF-dependent. It was therefore surprising to find that WT mice treated with CPAF immediately after PMA application also exhibited an approximately 50% loss of PMA-induced early ear thickness changes observed at 2 hrs (Fig 6B). This suggests a complex role for the PAF-R in regulating initial vasoactive edema changes in response to PMA.

While the above data suggests a complex role for the PAF-R in regulating initial PMA-induced inflammation, similar results were seen at 24 and 48 hrs, which represent the period in which inflammation is heavily dependent on inflammatory cell infiltrates. As with the 2 & 8 hr time points, *Ptafr*-/- mice exhibited reductions in PMA-induced ear thickness changes at 24 & 48 hrs. However, topical CPAF was also significantly suppressed the PMA-induced increases in ear thickness that occurred at 24 & 48 hrs (Fig 6B). That these effects are PAF-R dependent are seen by the complete loss of activity of CPAF in *Ptafr*-/- mice (Fig 6B). Thus, topical CPAF exerted complex, but PAF-R-dependent effects on PMA-induced edema and early inflammatory changes in mouse ears.

### Topical CPAF has no effect on PMA-induced inflammation in c-KitW-sh/W-sh mice

**Figure 7:**
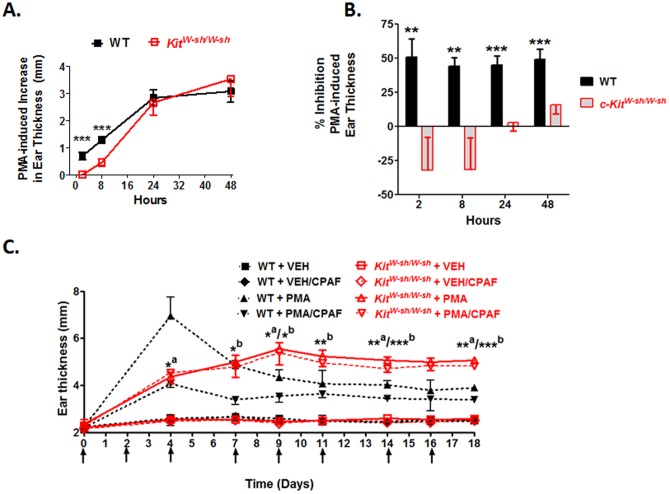
Kit^W-sh/W-sh^ mice exhibit a reduction in PMA-induced acute inflammation but an augmented chronic or sustained inflammatory response to multiple PMA applications. For all experiments in Fig 7A–C, ear thickness measurements were made just prior to reagent application as well as at the indicated time points up to 18 days. Results represent the mean and SEM of ear thickness (n = 4–14 mice per group). *, *p*<0.05; **, *p*<0.01; ***, *p*<0.01; 2-tailed *t*-test. *7A. Mast cell deficient mice have a blunted initial inflammatory response to a single application of PMA.* WT and c-*Kit^W-sh/W-sh^* mice were treated with VEH or PMA as described in Fig 4A&B. After subtracting the ear thickness at time 0 prior to PMA application, ear thickness changes were plotted (n = 12–14 mice/group). Ear thickness changes were significantly reduced in c-*Kit^W-sh/W-sh^* mice treated with PMA relative to WT + PMA mice at 2 and 8 hrs following PMA application. ***, *p*<0.001. *7B. Following a single application of PMA, topical CPAF treatment has no effect on PMA-induced ear thickness changes in c-Kit^W-sh/W-sh^ mice.* PMA-induced ear thickness changes were assessed in WT or c-*Kit^W-sh/W-sh^* mice treated with PMA or PMA + CPAF. After subtracting the ear thickness at time 0, the ability of CPAF to suppress PMA-induced ear thickness changes was calculated as a percentage inhibition of PMA-induced ear thickness increases. CPAF treatment resulted in a significant inhibition of PMA-induced ear thickness changes at all time points (see also fig 6B). CPAF treatment had no significant effect on PMA-induced ear thickness changes in c-*Kit^W-sh/W-sh^* mice (Wilcoxon Signed Rank Test). The percent inhibition of PMA-induced ear thickness changes by CPAF in WT mice was also significantly different than that seen in c-*Kit^W-sh/W-sh^* mice (**, *p*<0.01, ***, *p*<0.001; *t*-test). *7C. Topical CPAF is inactive in Kit^W-sh/W-sh^ mice while these mice also exhibit a reduction in acute inflammatory ear thickness changes observed in the first 4 days, but exhibit a significant increase in chronic sustained ear thickness changes.* Significant differences were noted at the indicated time points in WT mice treated with PMA relative to Kit^W-sh/W-sh^ mice treated with PMA (^a^) as well as in WT mice treated with PMA/CPAF relative to Kit^W-sh/W-sh^ mice treated with PMA/CPAF (^b^).

While mast cells are known mediators of PAF-induced edema reactions, PMA is also known to induce mast cell degranulation and mast cell deficient mice exhibit a marked decrease in both PMA-induced edema reactions and leukocyte infiltration [43]–[45]. Thus, we sought to determine whether CPAF would have any effect on PMA-induced inflammation in c-*Kit^W-sh/W-sh^* mice. In Fig 7A, we show that c-*Kit^W-sh/W-sh^* mice exhibit an impaired PMA-induced ear inflammation response at 2 and 8 hrs, consistent with a role in regulating early edema reactions. As in previous studies [43], this data suggests an important role for mast cells in the acute inflammatory effects of PMA on mouse skin. Given that loss of the PAF-R resulted in a similar loss in PMA-induced acute inflammatory effects, we determined whether c-*Kit^W-sh/W-sh^* mice were susceptible to CPAF-induced effects on inflammation. In Fig 7B, we show that the ability of topical CPAF to suppress PMA-induced ear thickness was absent in *Kit^W-sh/W-sh^* mice at all time points up to 48 hrs. Moreover, in the model of repetitive PMA applications (Fig 7C), *Kit^W-sh/W-sh^* mice exhibit a phenotype that mimics the phenotype seen in *Ptafr-/-* mice (see Figure S2 in File S1). Again, topical CPAF had no effect on PMA-induced chronic inflammation in c-*Kit^W-sh/W-sh^* mice (Fig 7C).

Finally, to verify that the changes in *Ptafr*-/- mice were not simply due to a loss of dermal mast cells, we next counted dermal mast cells in the ear skin of *Ptafr*-/- mice following 18 days of treatment with vehicle or PMA (Figure S4 in File S1). There was no significant difference in dermal mast cells in *Ptafr*-/- mice relative to WT mice in ears. This is in agreement with a recent study by another group [10]. However, PMA failed to induce a significant increase in mast cell numbers as previously reported [46]. This difference may be due to the fact that we used a shorter time period of PMA-induced inflammation than this previous report.

## Discussion

In this current study, we show that topical CPAF is pharmacologically active as it elicits a modest dose-dependent transient increase in ear thickness. Ear thickness changes peak at 2 hrs and are largely normalized at 8 hrs, consistent with transient edema formation. This is consistent with the well-known effects of PAF as a pro-inflammatory mediator. This pharmacological activity is further shown to be dependent on the PAF-R and is lost in c-Kit^W-sh/W-sh^ mice, suggesting a dependence on c-*Kit* as well. A major finding of our earlier study was that the sustained inflammation that occurs in response to repetitive PMA applications was increased in *Ptafr*-/- mice. While the pro-inflammatory effects of PAF are well known, this data suggests a counter regulatory role for the PAF-R in limiting the chronic or sustained inflammation associated with repeated PMA exposures. Consistent with this idea, our new studies demonstrate that co-administration of topical CPAF along with PMA results in a suppression of the sustained inflammatory response elicited by repetitive tumor promoter application. Since chronic inflammation is thought to promote carcinogenesis [35], [47]–[49], we previously suggested that the increased susceptibility to chemical carcinogenesis observed in *Ptafr*-/- mice may be due to anti-inflammatory effects. This is supported by our observations that topical CPAF suppresses DMBA/PMA-induced carcinogenesis, and that this chemopreventive activity correlates with a reduction in MPO activity.

In contrast to the effects of PAF-R activation on the sustained inflammatory response to multiple PMA applications, PAF-R signaling appears to exert complex pro-inflammatory and anti-inflammatory effects during the initial PMA-induced edema and inflammatory response observed in the first 48 hrs after a single PMA application. *Ptafr*-/- mice exhibited a reduction in ear thickness changes during the first 48 hrs following an initial PMA application. This would be consistent with PAF-R activation serving as a downstream mediator of PMA-induced acute inflammatory responses. This is indeed consistent with the known pro-inflammatory effects of PAF in regulating both edema and leukocyte recruitment [42], [43]. However, in our studies, topical CPAF is shown to consistently induce a paradoxical suppression of PMA-induced ear thickness at all time points following either a single PMA application and at later time points following multiple PMA treatments. The possibility that effects of exogenous CPAF are not PAF-R dependent is highly unlikely, as CPAF had no significant effect on PMA-induced inflammation at any time point in *Ptafr-/-* mice.

It is unclear how CPAF application (PAF-R activation) and loss of PAF signaling both result in reductions in PMA-induced edema &/or leukocyte infiltration at early time points following a single PMA application. This could indicate that exogenous CPAF exerts a supraphysiologic response at the receptor level that suppresses PMA-induced inflammatory signaling. It is known that some G-protein coupled receptors, particularly those coupled to Gi-alpha subunit signaling, can also activate Gbeta/gamma subunit signaling when receptors are activated at a higher molar level [50], [51]. Alternatively, it is possible that CPAF exerts effects on inflammation through a complex interplay between multiple cell types with counter-regulatory functions.

A second major finding of our study is that c-*Kit* is likely critical for our observed effects of CPAF on PMA-induced inflammation. Topical CPAF failed to elicit a response in c-*Kit^W-sh/W-sh^* mice at all time points following PMA application(s). Like PAF, mast cells have long been known to be pro-inflammatory mediators of type I hypersensitivity reactions [52]. More recent data indicates that mast cells exert complex effects on both innate and adaptive immune responses [26]–[28]. The resistance of mast cell deficient mice to the initial PMA-induced edema and inflammatory response is consistent with previous studies demonstrating that mast cell deficient mice exhibit a marked decrease in both PMA-induced edema reactions and leukocyte infiltration [43]–[45]. Moreover, our data suggests that mast cells may be necessary for the ability of CPAF to suppress the sustained inflammation observed following multiple PMA applications. It might be noted that a similar observation was noted in mice exposed to contact allergen or chronic low dose ultraviolet B [32]. In this study, mast cells limited the leukocyte infiltration, necrosis and epidermal hyperplasia that were associated with these insults [32]. Since mast cells can produce both pro-inflammatory and anti-inflammatory mediators [29], [31], it is possible that initially pro-inflammatory mast cells alter their secretory phenotype under conditions of repetitive chronic inflammatory stimuli resulting in a switch to anti-inflammatory mediator release. This idea is supported by our data that shows that c-*Kit^W-sh/W-sh^* mice exhibit a reduction in the initial PMA-induced inflammation, but exhibit enhanced sustained chronic inflammation with chronic repetitive PMA applications. The weak edema response noted by topical CPAF administration is consistent with data indicating that PAF fails to elicit histamine secretion from dermal mast cells from humans [8], [53], although PAF is a potent activator of histamine release from mast cells derived from peripheral blood or lung tissue [8]. In contrast, the ability of mast cells to promote immune suppression following UVB irradiation depends on a PAF-dependent mast cell migration to lymph nodes [10]. Finally, it should be noted that melanocytes are absent in c-*Kit^W-sh/W-sh^* mice. Moreover, c-*Kit^W-sh/W-sh^* mice also exhibit increased numbers of immature granulocytic myeloid derived suppressor cells (MDSCs) within the spleen [23]. Thus, it is possible that some or all of the effects that we observed in c-*Kit^W-sh/W-sh^* mice are independent of mast cells or require mast cell interaction with melanocytes or MDSCs.

While our findings demonstrate that c-*Kit^W- sh/W-sh^* mice exhibit enhanced inflammation following repeated PMA applications, a recent study demonstrated that *Kit^W^/Kit^W-v^* mice exhibited a decrease in inflammatory cell accumulation over a 7 week period following DMBA treatment and repeated PMA applications [54]. It is possible that this discrepancy could be accounted for by the absence of DMBA treatment in our studies. More likely, this report is consistent with the known reduction in differentiated bone marrow derived cells in *Kit^W^/Kit^W-v^* mice that is not observed in c-*Kit^W-sh/W-sh^* mice [24]. In support of this idea, *Kit^W^/Kit^W-v^* mice also exhibited a reduction in total CD45^+^, CD8^+^, F4/80^+^, and Gr-1^+^ cells prior to treatment [54].

While topical CPAF suppressed not only PMA-induced inflammation, it also suppressed NMSC formation following chemical carcinogenesis. Moreover, *Ptafr-/-* mice exhibit enhanced DMBA/PMA-induced tumorigenesis [7]. Given that c-*Kit* appears to be critical to the effects of PAF on PMA-induced inflammation, it would be of interest to perform future studies to determine whether c-*Kit^W-sh/W-sh^* mice exhibit increased tumorigenesis in response to chemical carcinogens or UVB treatments. It has been proposed that mast cells should promote skin cancer formation through multiple mechanisms, particularly their ability to suppress anti-tumor adaptive immune responses [29], [30]. In studies in which mast cells have been directly assessed in tumor models, mast cells are associated with both pro-tumor and anti-tumor activity in lymphoma and models of adenocarcinoma [55]–[60]. Given the above discussion, it should be noted that a recent study demonstrated that *Kit^W^/Kit^W-v^* mice exhibit a pronounced increase in DMBA/PMA induced tumor formation [54]. Since these mice exhibit changes in other bone marrow derived cell types, it is important to note that this tumor phenotype was reversed by mast cell reconstitution of the treated skin [54].

It has been proposed that PAF should promote skin cancer formation secondary to its ability to induce a systemic T-cell immunosuppression [61]. Similarly, our studies have shown that PAF-induced immunosuppression promotes the growth of syngeneic tumors in mice [19]. Thus, the ability of topical CPAF to suppress chemical carcinogenesis in mice would run counter to previous thoughts regarding the role of PAF in tumorigenesis. It is possible that both mast cells and PAF-R activation act to promote tumor formation via loss of immune surveillance mechanisms, but this is countered by their ability to suppress the promoting aspects of cutaneous inflammation. Thus, it may be that a tissue or context dependent balance between adaptive and innate immune system immunoregulation may determine the ultimate role of PAF in tumorigenesis. Alternatively, relatively high UVB doses are required to induce systemic immunosuppression [62]. This implies that relatively high systemic concentrations of PAF are necessary to induce systemic immunosuppression. Thus, it is possible that our relatively low doses of topical CPAF administration may readily elicit a suppression of local inflammation, but have minimal effects on systemic immunosuppression. The use of higher topical doses or systemic administration of CPAF in our current model may prove informative in future studies to address this idea. Finally, chronic repetitive PMA and/or CPAF applications could act to promote a change in specific mast cell mediator release, to promote an altered balance of phenotypically distinct mast cells (MMC or CTMC), or alter the microenvironmental cues that drive mast cell function. In any case, future studies are necessary to better address the relative role of PAF in altering both adaptive and innate immune responses in cutaneous carcinogenesis. Further work is also necessary to determine whether PAF-R signaling acts to suppress tumor formation in the more clinically relevant photocarcinogenesis model.

## Supporting Information

File S1 **Contains Figs S1-S4.**
(PDF)
