# Supplementary material for: Topical Application of a Platelet Activating Factor Receptor Agonist Suppresses Phorbol Ester-Induced Acute and Chronic Inflammation and Has Cancer Chemopreventive Activity in Mouse Skin
Source: PLoS One. 2014 Nov 6;9(11):e111608. doi: 10.1371/journal.pone.0111608 (PMC4222871; doi:10.1371/journal.pone.0111608)
Supplement: File S1 — Contains Figs S1-S4. (PDF) [file pone.0111608.s001.pdf]

Sahu *et al.*

Topical application of a platelet activating factor receptor agonist suppresses phorbol ester-induced acute and chronic inflammation and has cancer chemopreventive activity in mouse skin: Potential role of c-*Kit*

Supporting Information Contents

Figure S1
Figure S2
Figure S3A
Figure S3B

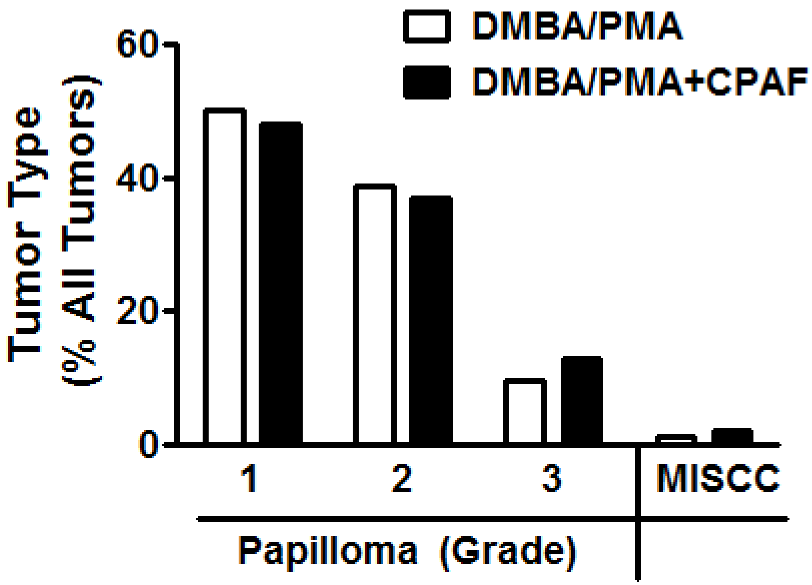

Figure S1: Topical treatment with CPAF has no significant effect on the distribution of grade 1-3 papillomas and microinvasive squamous cell carcinomas MISCC). Following a 25 week DMBA/PMA carcinogenesis study (with or without topical CPAF administration), tumors were removed and formalin fixed and paraffin-embedded. Following H&E staining, tumor type (grade 1-3 papilloma or microinvasive squamous cell carcinoma (MISCC)) was assessed as previously described [34].

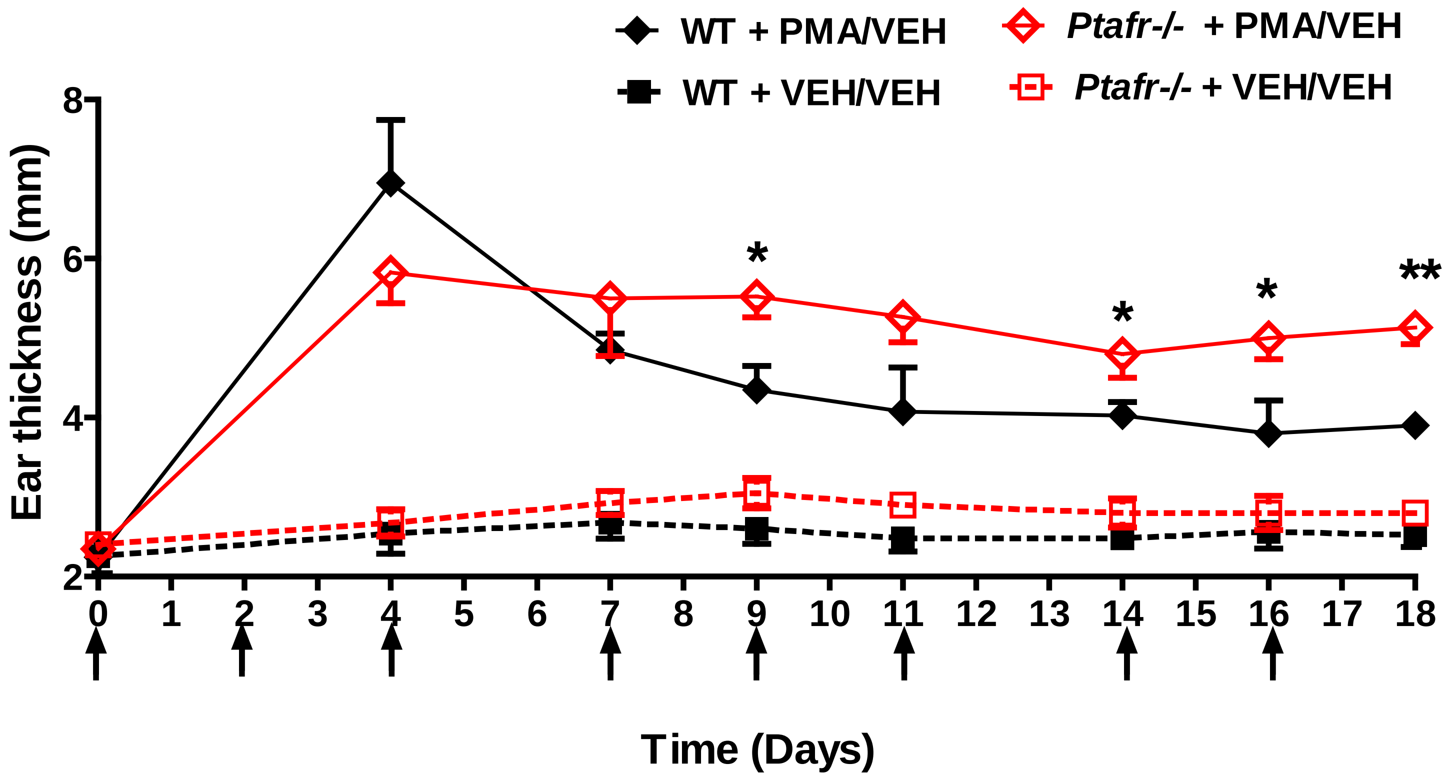

Figure S2: Repeat study validating previously published study [34] showing that *Ptafr*-/- exhibit increased chronic sustained ear thickness changes following thrice weekly PMA applications. Mean and SEM plotted (n=4-5 mice per group). *, p<0.05; **, p<0.01, 1-tailed t-test.

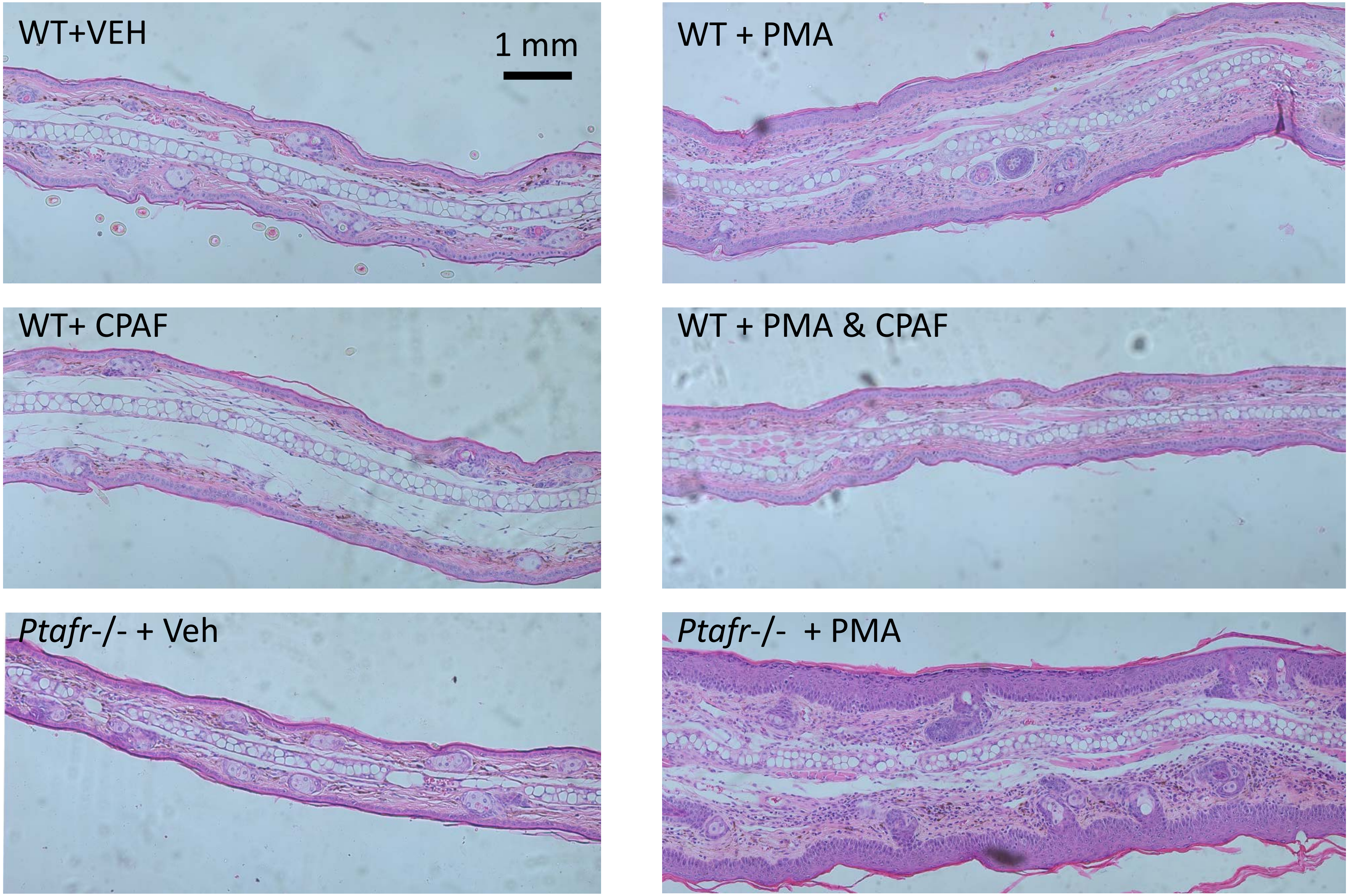

Figure S3A: Multiple low power (20X) images were taken of H&E stained ears following 18 days of treatment with PMA with/without CPAF treatment. The images were digitally stitched using Microsoft ICE software to provide a wide field of view.

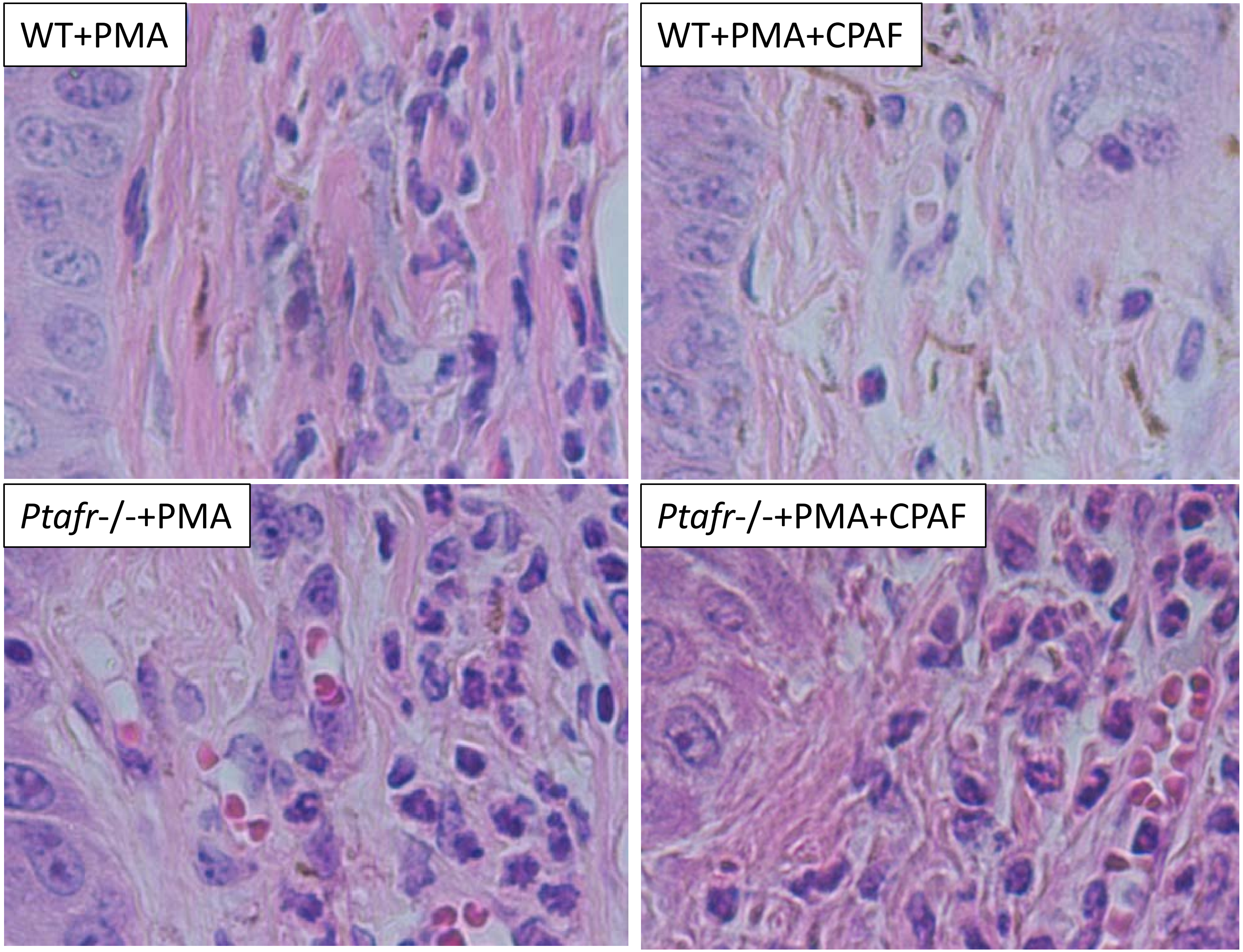

Figure S3B: Photomicrographs showing higher power images of PMA-treated ear skin from Fig 5.

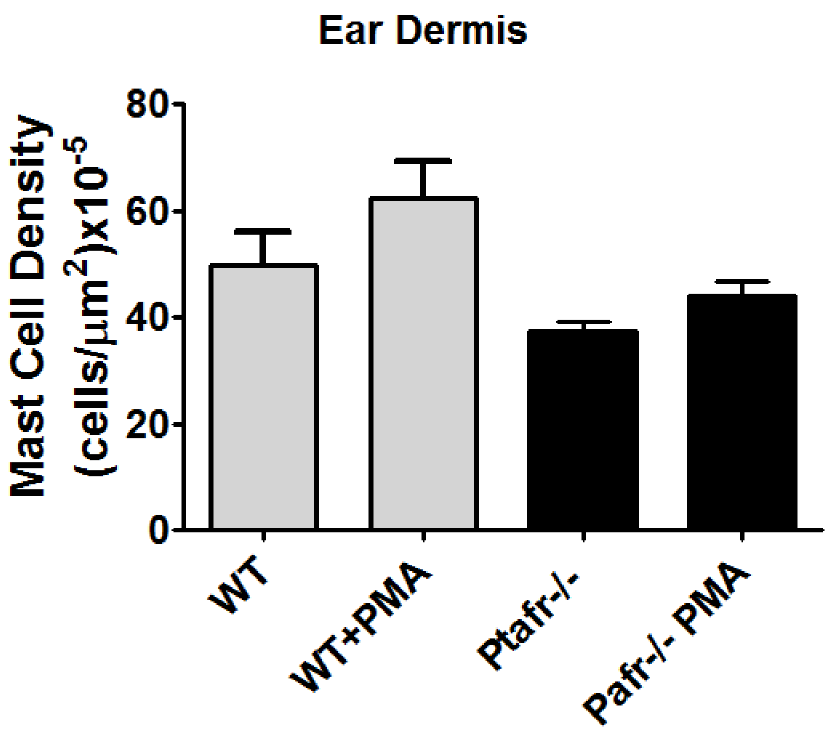

Figure S4: Mast cell density is not significantly altered by PAF-R status in mice treated with or without PMA for 18 days. Ear skin treated thrice weekly with PMA was formalin-fixed and paraffin embedded. Sections were stained using toluidine blue and mast cells were counted and normalized to the total dermal area.
